# Correlation Between Functional Scores and Objective Gait Parameters Following Rotating Hinge Knee Megaprosthesis Reconstruction

**DOI:** 10.1111/os.70300

**Published:** 2026-03-26

**Authors:** Meng‐Yu Chen, Ming‐Yong Gu, Sheng‐Rui Chu, Chong Li, Xue‐Fei Fu, Kuan Zhang, Ji‐Zhou Zeng, Yan‐Cheng Liu

**Affiliations:** ^1^ Department of Bone and Joint Surgery Beijing Luhe Hospital, Capital Medical University Beijing China; ^2^ Department of Bone and Soft Tissue Oncology Tianjin Hospital Tianjin China; ^3^ Department of Sports Medicine Third People's Hospital of Jinan City Jinan City Shandong Province China; ^4^ Tianjin Medical University Tianjin China; ^5^ Department of Orthopedics Provincial People's Hospital Hefei China; ^6^ School of Biomedical Engineering Capital Medical University Beijing China

**Keywords:** functional score, gait analysis, IDEEA, megaprosthesis

## Abstract

**Objective:**

Although the Musculoskeletal Tumor Society (MSTS) score, Toronto Extremity Salvage Score (TESS), and Knee Society Score (KSS) are widely used for postoperative functional evaluation in patients undergoing rotating hinge knee (RHK) megaprosthesis reconstruction, their accuracy in reflecting objective gait performance remains uncertain. The study aimed to analyze the correlations between these scoring systems and gait parameters.

**Methods:**

This retrospective study included 21 patients who underwent RHK between April 2023 and April 2025. At one‐year follow‐up, functional outcomes were assessed using MSTS, TESS, and KSS. Gait parameters were collected using the Intelligent Device for Energy Expenditure and Activity (IDEEA v3.1, MiniSun LLC). All functional assessments were completed on the same day as the gait analysis. Linear regression analyzed correlations between each scoring system and gait parameters. Friedman and Wilcoxon signed‐rank tests compared median coefficients of determination across scoring systems. Stepwise multiple linear regression was used to examine the relationships between the scoring system subitems and gait parameters.

**Results:**

Significant correlations were observed between all three scoring systems and multiple gait parameters after RHK (*p* < 0.05). The TESS had a significantly greater median *R* ^2^ than did the MSTS score and KSS (*p* < 0.05), especially for walking velocity (*R* ^2^ = 0.76), step length (*R* ^2^ = 0.75), initial contact phase (*R* ^2^ = 0.65), and stride length (*R* ^2^ = 0.58). Multiple linear regression analysis revealed that the MSTS “walking” and KSS “function” subitems independently or jointly predicted key gait parameters, including walking velocity, step length, and cadence (*R* ^2^ > 0.48).

**Conclusions:**

All three scoring systems showed correlations with multiple key gait parameters in patients who underwent RHK. However, TESS demonstrated stronger and more consistent correlations and therefore appeared to be a more representative score of gait recovery.

## Graphical abstract

Using a wearable gait system, we compared MSTS, TESS, and KSS with objective spatiotemporal metrics in patients after rotating‐hinge knee reconstruction. TESS showed the highest median *R* ^2^ and stronger correlations with walking velocity and step/stride length, outperforming MSTS and KSS.

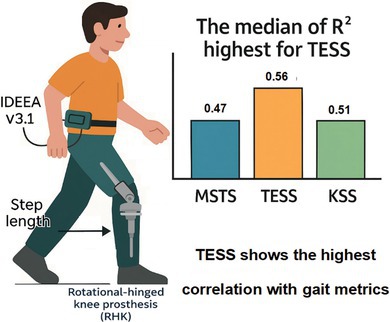

## 1 Introduction

Malignant bone tumors frequently affect the knee joint, with approximately 60% of osteosarcomas occurring in this region [1, 2]. Rotating hinge knee (RHK) megaprosthesis reconstruction is an important limb salvage strategy for malignant or locally aggressive knee bone tumors [3]. However, despite significant advances in tumor control and reconstruction, functional impairments such as decreased stride length, reduced walking speed, and gait asymmetry remain common after RHK [4, 5]. Accurate postoperative gait functional assessment is clinically essential for guiding individualized rehabilitation, forecasting long‐term outcomes, and informing prosthetic design optimization. Therefore, a simple, accurate tool for gait functional evaluation is urgently needed to better reflect postoperative recovery [6]. Gait analysis is an objective and quantitative tool for assessing motor function and is commonly used to evaluate walking ability and identify abnormal gait patterns. This type of analysis is widely applied in the clinic to assess treatment efficacy and rehabilitation progress and is considered the “gold standard” in motor function assessment [7]. In orthopedics, gait analysis has gradually become a valuable method for objectively assessing functional status before and after treatment [8]. Previous studies have demonstrated significant differences in critical gait parameters such as walking velocity, step length, stride length, cadence, gait cycle, stance phase, and single support time between patients who undergo RHK and healthy individuals, indicating that gait analysis has good sensitivity and reproducibility in capturing functional changes in patients who undergo RHK [9, 10]. Despite its advantages, gait analysis typically requires specialized laboratory settings, and its routine clinical application is restricted by high costs, operational complexity, and time‐consuming procedures [7]. Over the past decade, clinicians have used functional scoring tools in postoperative follow‐up due to their simplicity and ease of administration [11, 12]. The instruments most commonly used for clinical postoperative assessment include the Musculoskeletal Tumor Society (MSTS) score, the Toronto Extremity Salvage Score (TESS), and the Knee Society Score (KSS) [13, 14, 15]. Although research on functional evaluation in patients with megaprostheses following limb salvage surgery has increased, systematic comparisons between these scoring systems and objective gait parameters remain limited in the specific context of RHK reconstruction. Moreover, beyond simple correlation analyses, the relative explanatory performance of these instruments and the extent to which individual subitems capture clinically meaningful gait domains have not been sufficiently or clearly elucidated. Addressing these evidence gaps may support more evidence‐based selection and refinement of scoring tools in routine follow‐up and provide guidance for the design and implementation of future multicenter, longitudinal studies. The aims of this study were to: (i) quantify the associations between total MSTS, TESS, and KSS scores and objective gait parameters after RHK; (ii) compare the relative explanatory performance of these scoring systems using the coefficient of determination (*R* ^2^) to identify which score best reflects objective gait recovery; and (iii) examine whether specific subitems within the scoring systems independently predict key gait parameters, thereby informing a more targeted, clinically interpretable assessment framework and guiding future score refinement.

## 2 Materials and Methods

### 2.1 General Information

**FIGURE 1:**
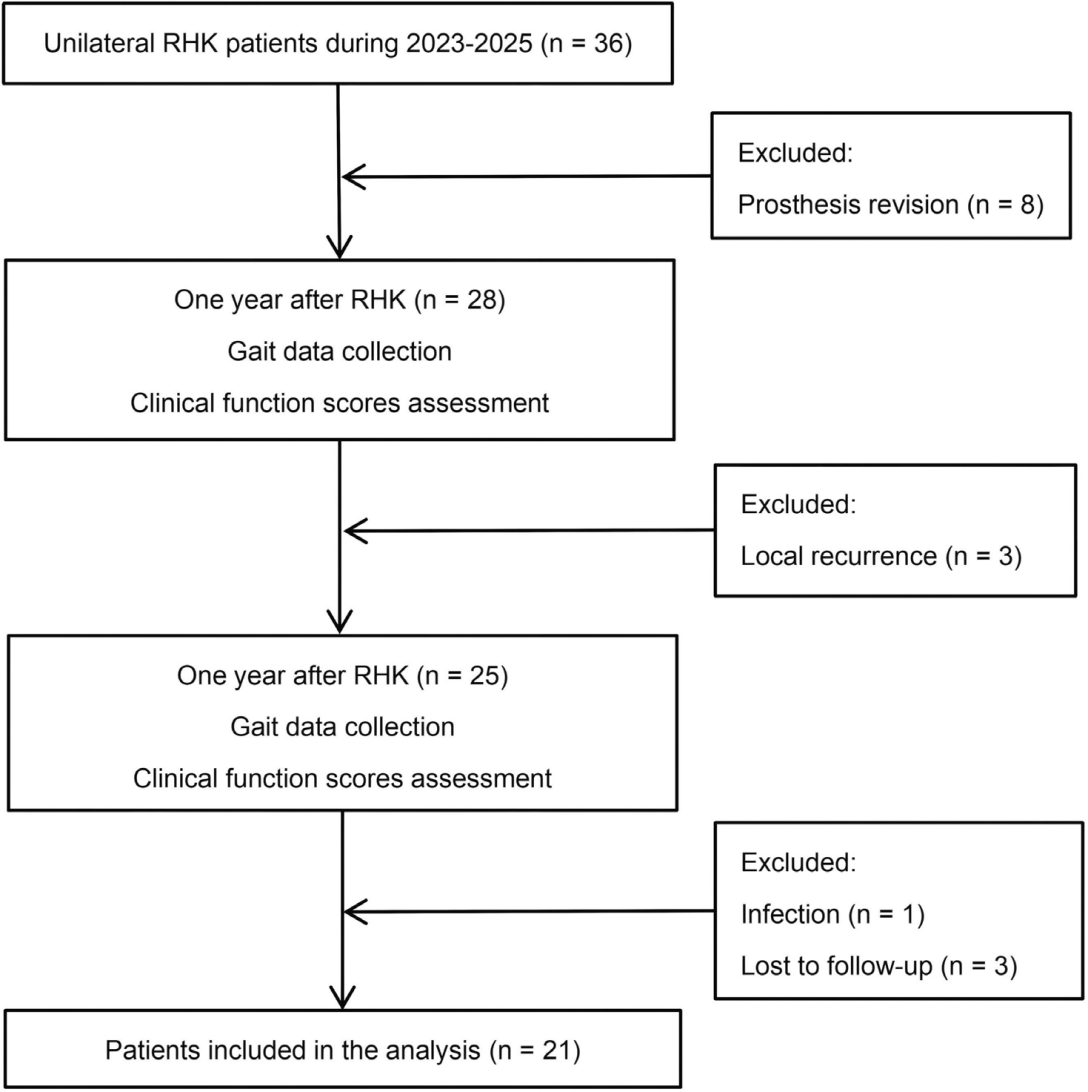
Flowchart of the inclusion and exclusion criteria.

In this retrospective cohort study, 36 patients who underwent unilateral RHK between April 2023 and April 2025 were screened. After the inclusion and exclusion criteria were applied, 21 patients were ultimately included (Table 1). The inclusion criteria were as follows: (1) a diagnosis of a primary bone tumor around the knee that was treated with resection and reconstruction using a rotating hinge prosthesis; (2) good postoperative wound healing with no local recurrence and a follow‐up duration of at least 12 months; (3) the ability to ambulate independently; the use of assistive devices (e.g., canes or walkers) was permitted; (4) an age ≤ 78 years; and (5) the absence of any comorbidities involving the hip or ankle joints and neurological disorders. The exclusion criteria were as follows: (1) other conditions that could affect gait (e.g., neuromuscular diseases or contralateral joint disorders); (2) a history of amputation, prosthesis revision, or tumor recurrence or a limb‐length discrepancy > 20 mm; and (3) incomplete data or loss to follow‐up. The flowchart of the enrollment process, assessment procedures, and exclusion process is presented (Figure 1). The Tianjin Hospital ethics committee approved all testing protocols (2020–084). All participants have signed an informed consent form.

**TABLE 1:** Baseline characteristics of the study subjects.

Patient	Gender	Age (years)	Height (m)	Weight (kg)	BMI (kg/m^2^)	PD	TL	Chemotherapy	Radiotherapy	Flap	Follow‐up (months)
1	M	53	1.76	89	28.73	Osteosarcoma	PT	No	Yes	MGMF	14
2	F	67	1.68	77	27.28	MFH	PT	No	No	MGMF	19
3	F	22	1.60	68	26.56	MBGCT	DF	No	No	No	19
4	M	21	1.68	66	23.38	Osteosarcoma	PT	No	Yes	MGMF	19
5	F	17	1.63	41	15.43	Osteosarcoma	PT	No	Yes	MGMF	15
6	F	34	1.64	67	24.91	MBGCT	DF	No	Yes	No	20
7	M	14	1.77	81	25.85	Osteosarcoma	DF	No	Yes	No	23
8	M	68	1.72	58	19.61	MBGCT	DF	No	no	no	23
9	M	17	1.70	58	20.07	Osteosarcoma	DF	No	Yes	No	12
10	M	62	1.70	70	24.22	MBGCT	DF	No	No	No	18
11	F	29	1.63	64.9	24.43	MBGCT	PT	No	yes	MGMF	13
12	M	19	1.83	85.8	25.62	Ewing sarcoma	PT	No	Yes	MGMF	19
13	M	68	1.71	71.7	24.52	MBGCT	PT	No	No	MGMF	17
14	F	26	1.70	63.6	22.01	Osteosarcoma	DF	No	Yes	No	17
15	F	52	1.62	56	21.34	Osteosarcoma	PT	No	yes	MGMF	13
16	F	75	1.58	55	22.03	Osteosarcoma	DF	No	No	No	17
17	M	59	1.72	65	21.97	Osteosarcoma	DF	No	No	No	14
18	M	58	1.74	70	23.12	MBGCT	DF	No	No	No	18
19	F	42	1.58	50.7	20.31	Osteosarcoma	DF	No	Yes	No	20
20	M	61	1.72	62	20.96	MBGCT	PT	No	No	MGMF	14
21	F	58	1.55	63	26.22	MBGCT	DF	No	No	No	14

*Note*: Continuous variables are expressed as the mean ± standard deviation (SD). Units: Age, years; Height, m; Weight, kg; BMI, kg/m^2^; Follow‐up, months.

Abbreviations: BMI, body mass index; DF, distal femur; F, female; M, male; MBGCT, malignant bone giant cell tumor; MFH, malignant fibrous histiocytoma; MGMF, medial gastrocnemius muscle flapPD, pathological diagnosis; PT, proximal tibia; SD, standard deviation; TL, tumor location.

### 2.2 Functional Scoring Systems

Three commonly used scoring systems were employed in this study: the MSTS scoring system, which is completed by clinicians, includes six subitems (pain, function, emotional acceptance, support, walking, and gait), each rated from zero to five points; the total possible score is 30 points, and higher scores indicate better function [11]. The TESS, a patient‐reported outcome measure, is designed for patients with lower‐extremity tumors who undergo limb‐salvage surgery. This scoring system comprises 30 items related to daily activities (such as dressing, performing household chores, and participating in outdoor activities), each scored from one to five points; the total possible score is 150 points, and higher scores indicate better function [16]. The KSS, which includes knee, pain, and function subscores, is applicable for both preoperative and postoperative functional assessments; the total score ranges from 0 to 200, and higher scores indicate better function [17]. All functional assessments were completed on the same day as the gait analysis.

### 2.3 Gait Analysis Device and Data Collection Method

**FIGURE 2:**
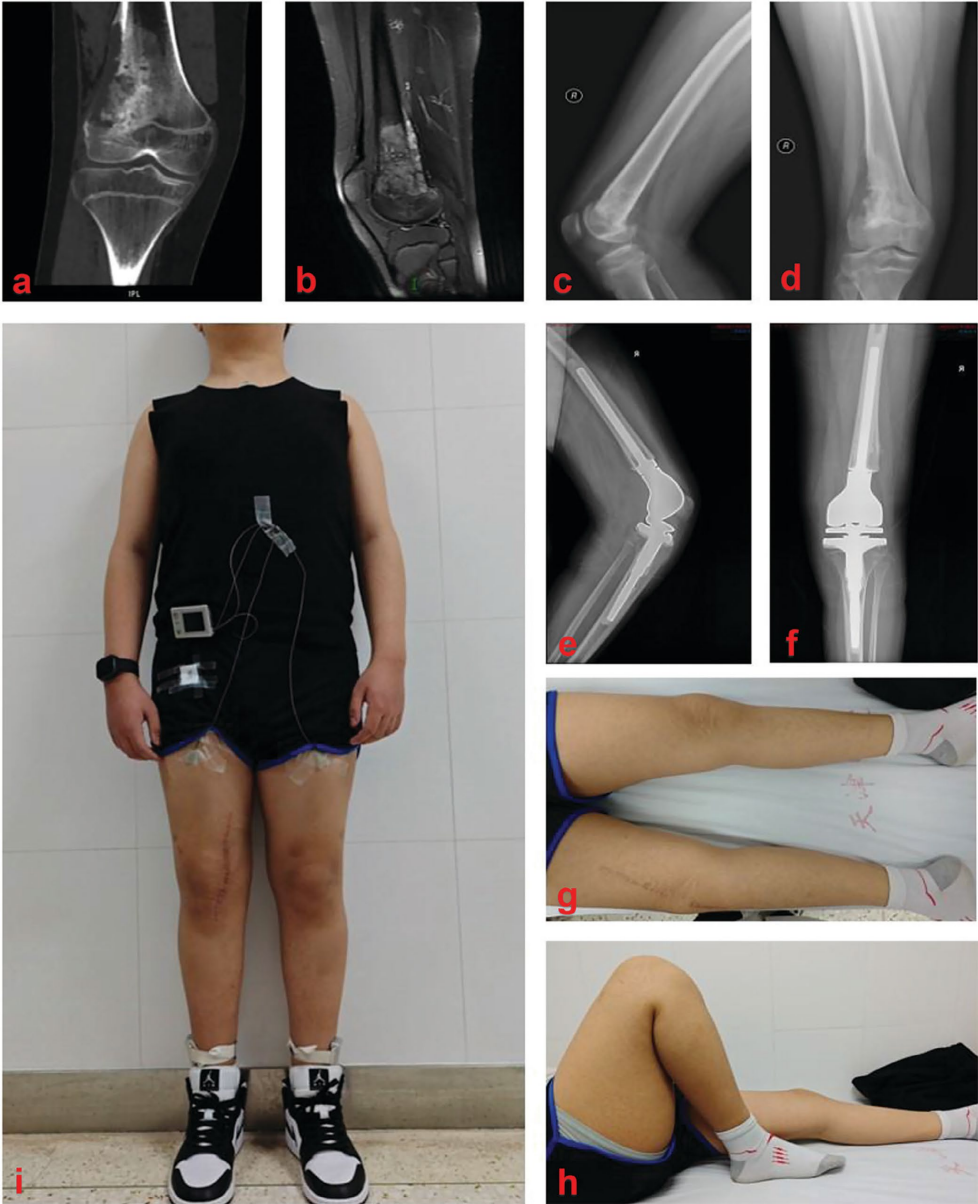
Representative case of an 11‐year‐old boy diagnosed with distal femoral osteosarcoma who underwent RHK (a, b) Preoperative CT and MRI scans of the affected knee; (c, d) Preoperative anteroposterior and lateral radiographs; (e, f) Postoperative anteroposterior and lateral radiographs; (g, h) One‐year follow‐up images showing satisfactory joint motion, stable fixation, and no evidence of prosthetic loosening; (i) Gait analysis via the IDEEA wearable system.

**FIGURE 3:**
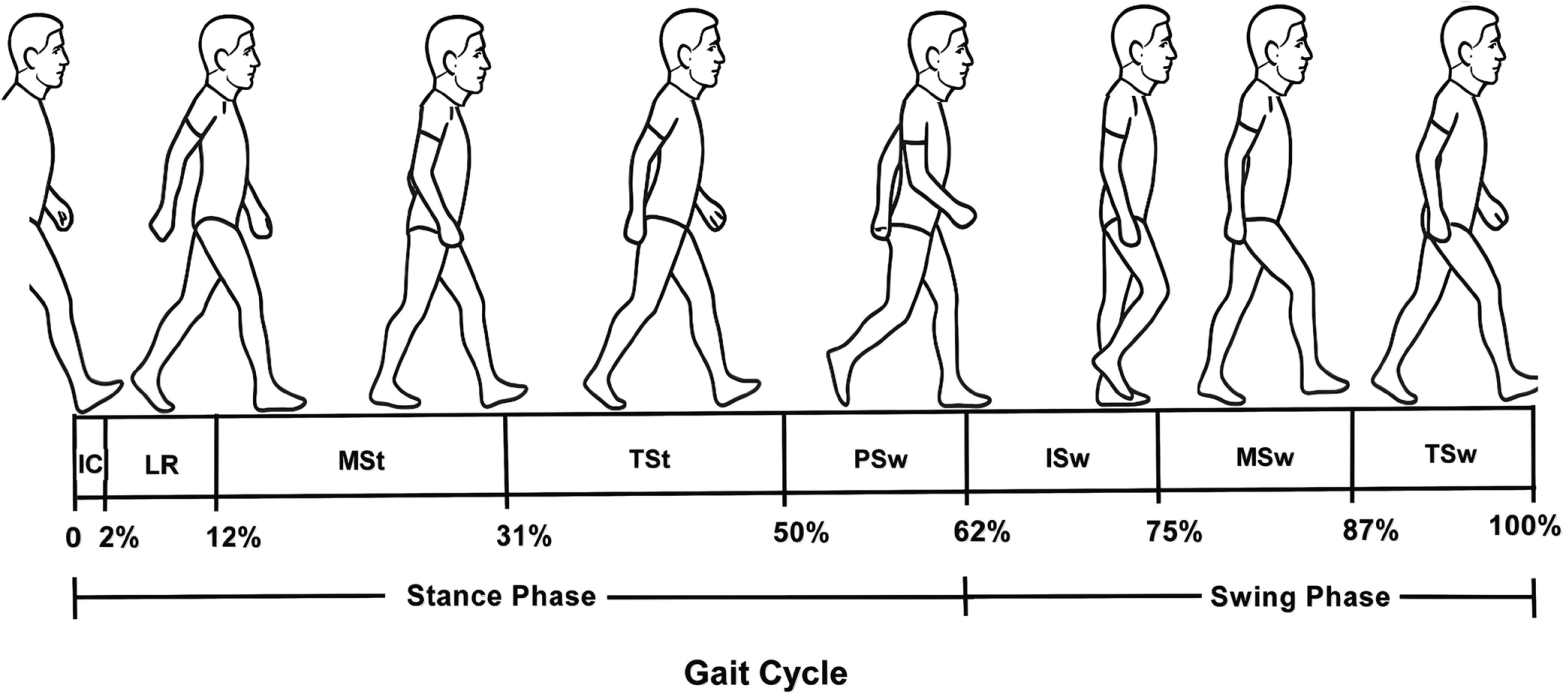
Schematic illustration of the human gait cycle. The gait cycle consists of two main phases: the stance phase and the swing phase. The stance phase includes initial contact (0%–2%), loading response (2%–12%), mid stance (12%–31%), terminal stance (31%–50%), and pre‐swing (50%–62%). The swing phase includes initial swing (62%–75%), mid swing (75%–87%), and terminal swing (87%–100%). Abbreviations: IC, initial contact; LR, loading response; MSt, mid stance; TSt, terminal stance; PSw, pre‐swing; ISw, initial swing; MSw, mid swing; TSw, terminal swing.

Gait analysis was conducted using the Intelligent Device for Energy Expenditure and Activity (IDEEA v3.1; MiniSun LLC, USA), a validated wearable system for monitoring ambulatory motion [18]. The system consists of a main recorder and five triaxial accelerometer sensors, which are affixed to the dorsal forefoot near the base of the fourth metatarsal on both feet, the mid‐anterior surface of both thighs, and the upper sternum (Figure 2i). The participants walked back and forth along a 30‐m flat indoor corridor at a self‐selected comfortable speed while wearing regular footwear. The gait cycle, also known as a stride, consists of two phases, the stance phase and the swing phase, which alternate between the two legs (Figure 3). Data from 20 consecutive gait cycles in the central portion of the trial were selected for analysis. The following gait parameters were extracted and analyzed in this study. The spatiotemporal parameters included step length, stride length, walking velocity, cadence, gait cycle, and stance time. The phase‐related parameters, expressed as a percentage of the gait cycle, included the initial contact phase, middle stance phase, loading response phase, initial double support phase, and single support phase. In addition, the pulling acceleration of the thigh was recorded as a dynamic parameter. All raw signals were processed using ActView 3.0 software (MiniSun LLC) to detect gait events and compute the corresponding gait parameters.

### 2.4 Surgical Procedure and Postoperative Rehabilitation

All surgeries were performed by a single experienced surgical team. Patients were placed in the supine position, and either an anteromedial or anterolateral incision extending from the mid‐thigh to the tibial tubercle was made depending on the tumor location. Intraoperatively, the femoral neurovascular bundle and the extensor mechanism were carefully preserved. Bone resection was carried out with an adequate distal margin, determined on the basis of comprehensive preoperative computed tomography (CT) and magnetic resonance imaging (MRI) assessments. The surgical procedure consisted of three main steps: tumor resection, prosthesis implantation, and soft tissue reconstruction. All patients underwent reconstruction using a rotating hinge knee prosthesis. A standardized rehabilitation protocol was implemented postoperatively for all patients, including pain management, knee range‐of‐motion exercises, muscle strengthening, and gait retraining [19]. On the basis of previous studies indicating that functional recovery generally stabilizes by 1 year after RHK, the 12‐month postoperative time point was selected for functional evaluation in this study [20]. A representative case illustrating the clinical management of an 11‐year‐old patient with distal femoral osteosarcoma (Figure 2). Preoperative imaging (Figure 2a–d) revealed a clearly defined lesion around the knee joint. The patient subsequently underwent RHK, and postoperative radiographs (Figure 2e,f) indicated good prosthesis positioning and stable fixation. At the 12‐month follow‐up (Figure 2g,h), the patient exhibited good joint mobility and no signs of prosthetic loosening, and gait measurements were conducted via the IDEEA system (Figure 2i).

### 2.5 Statistical Analysis

All the statistical analyses were performed using SPSS version 27.0 (IBM Corp., Chicago, IL, USA). Continuous variables are expressed as the means ± standard deviations (SDs) for normally distributed data or as medians with interquartile ranges (IQRs) for non‐normally distributed data. The Shapiro–Wilk test was used to assess normality. Pearson correlation analysis was applied to normally distributed variables, whereas Spearman's rank correlation was used for non‐normally distributed variables. Correlation‐strength categories were applied to the absolute coefficient, |*r*|, following Evans: < 0.20 very weak; 0.20–0.39 weak; 0.40–0.59 moderate; 0.60–0.79 strong; ≥ 0.80 very strong [21]. The Friedman test and Wilcoxon signed‐rank test were employed to compare the median coefficients of determination (*R* ^2^) among the three scoring systems. For gait parameters that were significantly correlated with the total scores, stepwise multiple linear regression analysis was conducted to identify independent predictive subitems within each scoring system. All the statistical tests were two‐sided, and *p* values < 0.05 were considered to indicate statistical significance.

## 3 Results

### 3.1 Correlations Between Functional Scores and Gait Parameters

**FIGURE 4:**
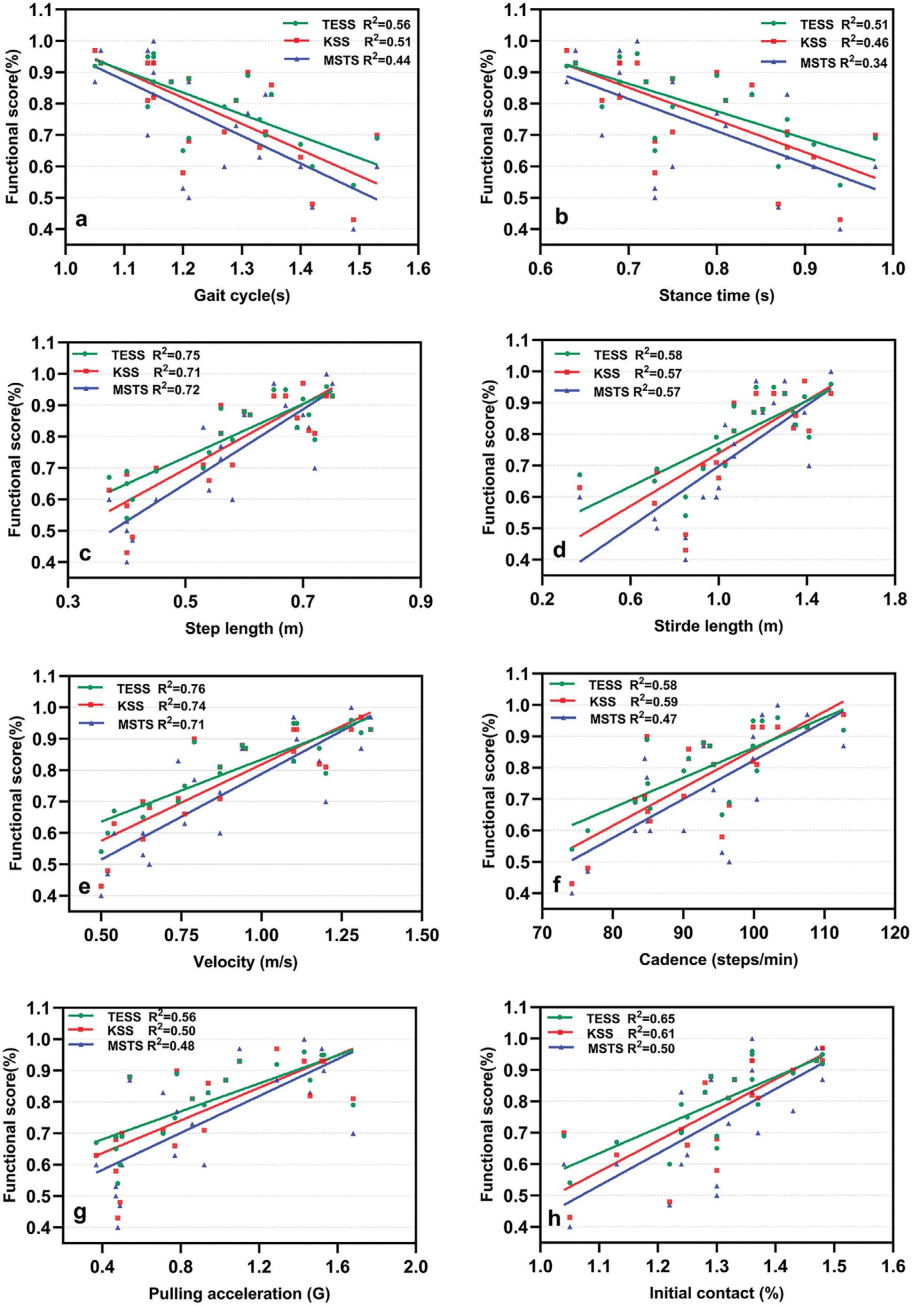
Pearson correlation analysis between functional scores and gait parameters after RHK. Significant correlations were observed between functional scores and the following gait parameters: (a) gait cycle, (b) stance time, (c) step length, (d) stride length, (e) walking velocity, (f) cadence, (g) pulling acceleration, and (h) initial contact (*p* < 0.05). Abbreviations: MSTS, musculoskeletal tumor society score; TESS, Toronto extremity salvage score; KSS, knee society score.

The MSTS score was very strongly correlated with step length (*r* = 0.85; *R* ^2^ = 0.72) and walking velocity (*r* = 0.84; *R* ^2^ = 0.71). It was strongly correlated with stride length (*r* = 0.75; *R* ^2^ = 0.57), the initial contact phase (*r* = 0.71; R^2^ = 0.50), pulling acceleration (*r* = 0.69; R^2^ = 0.48), cadence (*r* = 0.68; *R* ^2^ = 0.47), and gait cycle (*r* = −0.66; R^2^ = 0.44). Moderate correlations were observed between the MSTS score and the middle stance phase, stance time, and the initial double‐support phase. No significant correlation was observed with the single support phase or loading response phase. The TESS score exhibited very strong correlations with walking velocity (*r* = 0.87; *R* ^2^ = 0.76), step length (*r* = 0.86; *R* ^2^ = 0.75), and initial contact phase (*r* = 0.80; *R* ^2^ = 0.65). It was strongly correlated with cadence (*r* = 0.76; *R* ^2^ = 0.58), stride length (*r* = 0.76; *R* ^2^ = 0.58), gait cycle (*r* = −0.75; *R* ^2^ = 0.56), pulling acceleration (*r* = 0.75; *R* ^2^ = 0.56), stance time (*r* = −0.72; *R* ^2^ = 0.51), and the middle stance phase (*r* = −0.62; *R* ^2^ = 0.38), whereas it was moderately correlated with the initial double support phase and loading response phase. No significant correlation was found with the single support phase. The KSS score was very strongly correlated with walking velocity (*r* = 0.86; *R* ^2^ = 0.74) and step length (*r* = 0.84; *R* ^2^ = 0.71). It was strongly correlated with initial contact phase (*r* = 0.78; *R* ^2^ = 0.61), cadence (*r* = 0.76; *R* ^2^ = 0.59), stride length (*r* = 0.75; *R* ^2^ = 0.57), gait cycle (r = −0.72; *R* ^2^ = 0.51), pulling acceleration (r = 0.71; *R* ^2^ = 0.50), and stance time (*r* = −0.67; *R* ^2^ = 0.46), whereas it was moderately correlated with the middle stance phase, initial double support phase, and loading response phase (Figure 4). Similar to the other two scoring systems, the KSS showed no significant correlation with the single support phase (Table 2).

**TABLE 2:** Correlation coefficients between clinical scores and gait parameters.

Gait parameter	MSTS (*r*/*R* ^2^)	TESS (*r*/*R* ^2^)	KSS (*r*/*R* ^2^)
Gait cycle(s)	(−0.66/0.44)**	(−0.75/0.56)**	(−0.72/0.51)**
Walking velocity (m/s)	(0.84/0.71)**	(0.87/0.76)**	(0.86/0.74)**
Cadence (steps/min)	(0.68/0.47)*	(0.76/0.58)**	(0.76/0.59)**
Step length (m)	(0.85/0.72)**	(0.86/0.75)**	(0.84/0.71)**
Stride length (m)	(0.75/0.57)**	(0.76/0.58)**	(0.75/0.57)**
Stance time (s)	(−0.58/0.34)*	(−0.72/0.51)**	(−0.67/0.46)**
Single support phase (%)	NA	NA	NA
Pulling acceleration (G)	(0.69/0.48)**	(0.75/0.56)**	(0.71/0.50)**
Initial contact (%)	(0.71/0.50)**	(0.80/0.65)**	(0.78/0.61)**
Initial double support phase (%)	(0.46/0.21)*	(0.53/0.28)*	(0.5/0.25)*
Loading response (%)	NA	(0.49/0.24)*	(0.47/0.22)*
Middle stance (%)	(−0.58/0.34)**	(−0.62/0.38)**	(−0.51/0.26)*

*Note*: r, pearson correlation coefficient; *R* ^2^, coefficient of determination; *p* < 0.05 and *p* < 0.01 are indicated by * and **, respectively. NA indicates a non‐significant correlation (*p* > 0.05).

Abbreviations: KSS, knee society score; MSTS, musculoskeletal tumor society score; TESS, Toronto extremity salvage score.

### 3.2 Comparisons of the Coefficient of Determination (R^2^ ) Among the Scoring Systems

**FIGURE 5:**
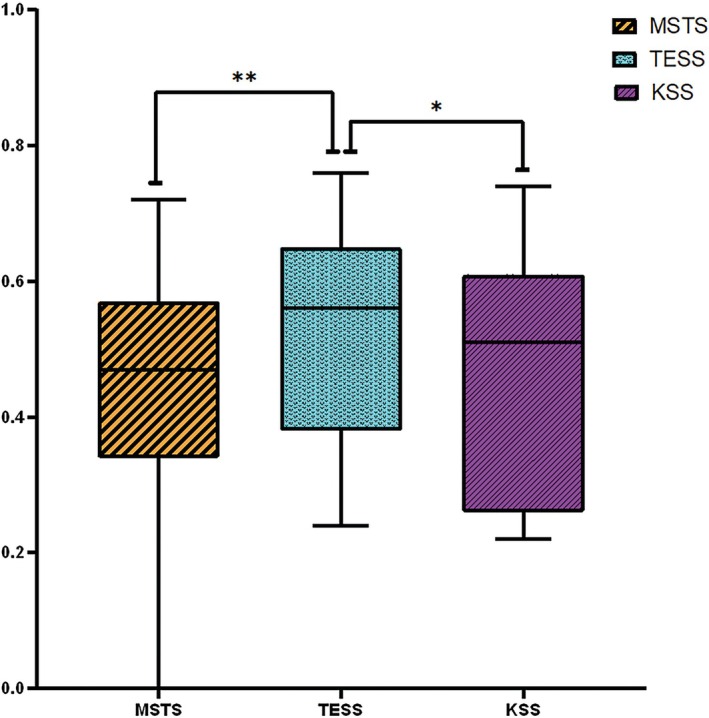
The distribution of median *R* ^2^ values derived from linear regression analyses between MSTS, TESS, and KSS scores and gait parameters. TESS demonstrated the highest median *R* ^2^ value, significantly exceeding those of MSTS and KSS. Statistical significance was assessed using the Friedman test followed by Wilcoxon post hoc comparisons. *p* < 0.05 and *p* < 0.01 are indicated by * and **, respectively.

The TESS exhibited the highest median coefficient of determination (*R* ^2^ = 0.56; IQR: 0.38–0.65), followed by the KSS (*R* ^2^ = 0.51; IQR: 0.26–0.61) and MSTS score (*R* ^2^ = 0.47; IQR: 0.34–0.57) (Table 3). The Friedman test revealed a statistically significant difference in the median R^2^ values among the three scoring systems (*p* = 0.0003). Post hoc Wilcoxon signed‐rank tests revealed that the TESS had significantly higher *R* ^2^ values than both the KSS and MSTS score (*p* < 0.05). In contrast, the difference between the MSTS score and KSS did not reach statistical significance (*p* = 0.407) (Figure 5).

**TABLE 3:** Comparison of the median *R* ^2^ among clinical scoring systems.

Comparison	MSTS	TESS	KSS	Test	*p*
The median of *R* ^2^ (IQR)	0.47 (0.34–0.57)	0.56 (0.38–0.65)	0.51 (0.26–0.61)	Friedman	0.0003**
MSTS versus KSS	—	—	—	Wilcoxon	0.407
MSTS versus TESS	—	—	—	Wilcoxon	0.00024**
KSS versus TESS	—			Wilcoxon	0.043*

*Note*: Data are presented as median *R* ^2^ values with interquartile ranges (IQR). The Friedman test was used to assess overall differences in the predictive performance of the three scoring systems. Wilcoxon signed‐rank tests were conducted for post hoc pairwise comparisons. *p* < 0.05 and *p* < 0.01 are indicated by * and **, respectively. “–” indicates not applicable.

Abbreviations: KSS, knee society score; MSTS, musculoskeletal tumor society score; TESS, Toronto extremity salvage score.

### 3.3 Multiple Linear Regression Analysis of the Scoring Subitems and Gait Parameters

As summarized in Table 4, among the six subitems of the MSTS score, the “walking” subitem independently predicted walking velocity (*R* ^2^ = 0.66), stance time (*R* ^2^ = 0.52), and pulling acceleration (*R* ^2^ = 0.53). Other gait parameters were jointly predicted by combinations of subitems: step length was jointly predicted by the “walking” and “pain” subitems (*R* ^2^ = 0.80), cadence was jointly predicted by “walking” and “support” (*R* ^2^ = 0.67), and the gait cycle was jointly predicted by “walking”, “support”, and “pain” (*R* ^2^ = 0.73). Among the three subitems of the KSS, the “function” subitem independently predicted walking velocity (*R* ^22^ = 0.67), cadence (*R* ^2^ = 0.63), step length (*R* ^22^ = 0.48), gait cycle (*R* ^2^ = 0.48), and stance time (*R* ^2^ = 0.46). Additionally, the “pain” subitem independently predicted pulling acceleration (*R* ^22^ = 0.43). Owing to concerns of multicollinearity, the TESS was excluded from the regression model.

**TABLE 4:** Regression analyses between the MSTS and KSS subitems and gait parameters.

Gait parameter	MSTS subitem	*p*	*R* ^2^	KSS subitem	*p*	*R* ^2^
Gait cycle(s)	Walking	< 0.001	0.73	Function	< 0.001	0.48
	Support	0.002	—	—	—	—
	Pain	0.049	—	—	—	—
Stance time (s)	Walking	< 0.001	0.52	Function	< 0.001	0.46
Step length (m)	Walking	< 0.001	0.80	Function	< 0.001	0.48
	Pain	0.007	—	—	—	—
Cadence (steps/min)	Walking	< 0.001	0.67	Function	< 0.001	0.63
	Support	< 0.001	—	—	—	—
Walking velocity (m/s)	Walking	< 0.001	0.66	Function	< 0.001	0.67
Pulling acceleration (G)	Walking	< 0.001	0.53	Pain	0.001	0.43

*Note: R* ^2^ denotes the coefficient of determination. A *p* value < 0.05 was considered statistically significant. The MSTS subitems include walking, gait, pain, function, psychological acceptance, and support. The KSS subitems comprise function score, pain score, and knee score. The symbol “—” indicates data not applicable.

Abbreviations: KSS, knee society score; MSTS, musculoskeletal tumor society score.

## 4 Discussion

To our knowledge, this study is the first to demonstrate differences among three widely used functional scoring systems in their ability to reflect postoperative gait function in patients who underwent RHK. Overall, MSTS, TESS, and KSS were significantly associated with multiple key gait parameters. Among them, TESS showed more pronounced associations with several key spatiotemporal gait metrics, particularly walking velocity and cadence. While the MSTS score and KSS demonstrated comparable overall validity, specific subitems, such as the MSTS “walking” subitem and the KSS “function” subitem, exhibited strong predictive value, suggesting the necessity of both global and item‐level functional evaluation following RHK.

### 4.1 Correlations Between Functional Scores and Gait Parameters

#### 4.1.1 Correlation Between the MSTS and Gait Parameters

Previous research on functional outcomes in patients with tumor endoprostheses has largely reported postoperative changes in a single scoring instrument, with only a few studies examining the associations between these scores and objective measures of activity. The MSTS score, as a motor task‐oriented tool, primarily reflects generalized functional capacity but lacks the sensitivity to detect subtle aspects of gait dynamics, such as the single support phase or loading response phase [22]. This observation is consistent with the findings reported by Furtado et al. [23]. Conversely, Rompen et al. failed to detect any significant correlation between the MSTS score and gait parameters, possibly owing to methodological heterogeneity in prosthesis types and variations in follow‐up duration across their cohort [24]. Sugiura et al. reported a significant correlation between the MSTS score and daily step count [25], whereas Rosenbaum et al. reported no association [26]. Both studies had similar limitations in that they excluded critical gait parameters such as walking velocity and cadence, thus restricting their ability to accurately evaluate the external validity of the MSTS score.

#### 4.1.2 Correlation Between the KSS and Gait Parameters

The KSS, which was originally developed for patients undergoing primary total knee arthroplasty, consists of clinical and functional components [17]. Freijo et al. reported preoperative correlations between the KSS score and gait parameters—such as walking velocity and stride length—in patients with knee osteoarthritis (OA), although the strength of these associations was generally moderate to low [27]. Liebensteiner et al. reported that the preoperative KSS was correlated with gait parameters in OA patients, but this association weakened significantly postoperatively [28]. This discrepancy might result from OA patients having a preoperative walking ability greater than that of patients who undergo RHK, limiting postoperative improvement potential in gait metrics despite noticeable score improvements, thus highlighting the variability in score validity across differing clinical contexts [29, 30]. Our study extends these findings to patients who undergo RHK and reveals that the KSS score is significantly correlated with most gait parameters. These findings suggest a strong correlation between the KSS and postoperative gait characteristics in patients undergoing RHK.

#### 4.1.3 Correlation Between the TESS and Gait Parameters

The TESS, in contrast, is a patient‐reported outcome measure (PROM) that captures the subjective perception of daily function. Although previous studies have predominantly utilized the TESS to evaluate postoperative activity levels in patients with musculoskeletal tumors, research exploring its association with objective gait parameters remains limited [31]. Rosenbaum et al. reported correlations between the TESS and the daily step count; however, they did not analyze key spatiotemporal gait parameters (such as walking velocity and cadence) in their study, thus limiting the extent to which their findings can be generalized and interpreted [26]. Our findings provide novel evidence that the TESS strongly correlates with specific spatiotemporal parameters, including step length and walking velocity, in patients who undergo RHK. Notably, none of the three functional scores demonstrated significant correlation with the single support phase. Occupying approximately 40% of the gait cycle, this phase is critically associated with dynamic postural stability and neuromuscular control. Studies have shown that a reduction in single support phase duration is frequently indicative of lower limb dysfunction, particularly among individuals with knee osteoarthritis [32]. Moreover, the single support phase has been identified as a sensitive indicator for gait symmetry, proprioceptive acuity, and central balance regulation [33]. The inability of MSTS, TESS, and KSS to reflect this critical spatiotemporal gait parameter may stem from their predominant reliance on patients' subjective perceptions of pain, mobility, and daily functional capacity, with limited consideration given to objective measures of neuromuscular control [34].

### 4.2 Comparisons of the Coefficient of Determination (R^2^ ) Among the Scoring Systems

The superior performance of the TESS may be attributed to its holistic design, which captures patients' self‐perceived ability to perform daily activities [16]. Unlike clinician‐assessed scores (MSTS score, KSS), the TESS integrates functional independence, endurance, and coordination—factors closely linked to ambulatory performance [35]. On the other hand, the MSTS score and KSS tend to emphasize task success (e.g., walking, climbing stairs) and may fail to capture more subtle elements, such as compensatory gait patterns or neuromuscular coordination [36].

### 4.3 Multiple Linear Regression Analysis of the Scoring Subitems and Gait Parameters

Moreover, our analysis demonstrated that the “function” subitem of the KSS has independent predictive value for key gait parameters such as walking velocity and cadence. The MSTS “walking” subitem also demonstrated independent predictive value for key gait outcomes, including walking velocity and pulling acceleration. Owing to the large number of subitems in the TESS posing a risk of multicollinearity and model overfitting, the TESS was not included in the multiple regression analysis. Nonetheless, this exclusion does not diminish its overall assessment validity, as different analytical approaches serve different purposes—univariate regression reflects overall explanatory power, whereas multiple regression identifies the most predictive subitems within a scoring system [37]. These results suggest that even in the context of oncologic reconstruction, the KSS may still provide clinically interpretable supplementary information at the item level. Meanwhile, the independent predictive value observed for the MSTS “walking” subitem and the KSS “function” subitem indicates that a small number of core items within broader scoring systems may retain high sensitivity and practical utility, providing a basis for developing streamlined assessment tools tailored for gait prediction in the future. Given that existing instruments may insufficiently capture balance and dynamic stability domains, future scoring frameworks could consider incorporating additional balance focused items or combining functional scores with brief balance tests, such as the Timed Up and Go test or a one leg standing balance test [38, 39]. Overall, the TESS appears to better capture gait related functional limitations and may be particularly suitable for outpatient follow up or resource limited settings without access to gait laboratories [31]. For clinicians who continue to use the MSTS or KSS, interpretation should place particular emphasis on the MSTS “walking” item and the KSS “function” item to improve the specificity and clinical interpretability of postoperative functional assessment.

### 4.4 Limitations and Strengths

This study focuses on a clinically important RHK population. Postoperative functional assessment in this group is challenging, and the available evidence remains limited. Using objective gait parameters as a reference, we systematically evaluated the associations between commonly used clinical functional scores and real world walking performance. We further compared the relative explanatory performance of different instruments and conducted item level predictive analyses. Overall, this study provides an evidence base to support the selection and optimization of functional assessment tools after RHK and offers direction for future development of clinically useful postoperative assessment tools. There are several limitations in this study. First, the single‐centre retrospective design and modest sample size may limit the generalizability of our findings. Second, our retrospective cross‐sectional study cannot establish causal relationships or dynamic trajectories. Finally, although tumor location, resection length, extent of soft‐tissue sacrifice, and surgical approach may affect function and gait, the modest sample size led us to avoid over‐stratification to prevent unstable estimates; therefore, these factors were not stratified or analyzed separately. Future research should consider conducting multi‐center, prospective studies with larger sample sizes, stratified by tumor type, location, resection length, soft‐tissue sacrifice, and surgical approach, to validate and expand upon the current findings.

## 5 Conclusion

In this study, all three scoring systems showed correlations with multiple key gait parameters in patients who underwent RHK. However, TESS demonstrated stronger and more consistent correlations and therefore appeared to be a more representative score of gait recovery.

## Author Contributions

All authors contributed to the study design and interpretation. Data collection and preliminary analysis were carried out by Meng‐Yu Chen, Ming‐Yong Gu, and Sheng‐Rui Chu. The initial draft of the manuscript was written by Meng‐Yu Chen and Ming‐Yong Gu. Sheng‐Rui Chu, Chong Li, and Xue‐Fei Fu contributed to data interpretation and critical revision of the manuscript. Kuan Zhang, Ji‐Zhou Zeng, Ming‐Yong Gu, and Yan‐Cheng Liu provided overall supervision and were responsible for protocol development and manuscript refinement. All authors reviewed and approved the final version of the manuscript.

## Funding

This work was supported by Tongzhou District Science and Technology Foundation Beijing, China, KJ2023SS001, Science and Technology Program of Jinan Health Commission, 2023–2–62, Wu Jieping Foundation, 320.6750.2022‐18‐49.

## Disclosure

The authors have nothing to report.

## Conflicts of Interest

The authors declare no conflicts of interest.

## Data Availability

The data that support the findings of this study are available on request from the corresponding author. The data are not publicly available due to privacy or ethical restrictions.
